# Efficacy of Embolization in Acquired Uterine Vascular Malformations: An Experience in Tertiary Care Centre in India

**DOI:** 10.1055/s-0043-1770092

**Published:** 2023-07-21

**Authors:** Vineel Inampudi, Sunanda Nimmalapudi

**Affiliations:** 1 Department of Radiodiagnosis, Sri Venkateswara Medical College, Tirupati, Andhra Pradesh, India

**Keywords:** Uterine arteriovenous malformations, Uterine artery embolization, Uterus, Endovascular, Histoacryl glue

## Abstract

**Objective:** To determine the efficacy of Uterine Artery Embolization in patients with bleeding acquired uterine arteriovenous malformations (AVMs).

**Methods:** A prospective review of all patients who underwent Uterine Artery Embolization at our institution between July 2015 and April 2022 was performed. 225 patients were diagnosed with a uterine vascular malformation on doppler and corresponding MRI imaging. All patients underwent transcatheter embolization of the uterine arteries. Embolic agents in the 375 procedures included Histoacryl glue only (n = 326), polyvinyl alcohol (PVA) particles and Histoacryl glue (n = 29), PVA particles (n = 5), Gelfoam (n = 5), coils (n = 4), PVA particles and coils (n = 3), Histoacryl glue and Gelfoam (n = 2), and Histoacryl glue and coils (n = 1).

**Results:** A total of 375 embolization procedures were performed in 225 patients. 90 patients required repeat embolization for recurrence of bleeding. The technical success rate of embolization was 100%. The clinical success rate was 92%: bleeding was controlled in 222 of 225 patients and three patients underwent a hysterectomy. 60 of the 225 patients had uneventful intrauterine pregnancies carried to term. The 210 patients who underwent successful embolization had no recurrence of bleeding at a median follow-up of 53 months (range, 5-122 months) after treatment. 15 patients were eventually lost to follow-up. One minor complication (0.4%) of non-flow-limiting dissection of the internal iliac artery occurred.

**Conclusion:** Uterine Artery Embolization is a safe, effective, minimally invasive method to treat uterine AVMs with long-term efficacy, which can provide the preservation of fertility.

## Introduction

Arteriovenous malformations (AVMs) can be found anywhere in the vascular system, including the uterus. Uterine AVMs consisting of an abnormal connection between an artery and a vein. They usually occur in women of reproductive age, but are very rare after menopause. Uterine AVM is a rare but potentially severe cause of genital haemorrhage that can be life-threatening. 1 Unlike other conditions, curettage is not therapeutic and may aggravate the bleeding. 2 3 Uterine AVMs are most commonly observed after pregnancy that occurs in women with a past history of induced abortion, curettage, uterine surgery, caesarean section, or diethylstilbestrol exposure. 4 5 6 7 8 The clinical diagnosis of uterine AVM is often difficult. However, correct diagnosis can be reliably made using imaging by doppler ultrasonography (USG). It is a low cost and non-invasive procedure. Other imaging methods including computed tomography (CT) and magnetic resonance imaging (MRI) may be used to complement the diagnosis in difficult cases and are used to determine its size, extent and vascularity, and the involvement of adjacent organs. Traditionally uterine AVMs have been surgically treated with hysterectomy or uterine artery ligation. More recently, endovascular treatment has proven to be an effective alternative. 9 Because most uterine AVMs are diagnosed in women of childbearing age, the recent development of curative embolization therapy should reduce the indications for hysterectomy and preserve fertility. 10 For uterine AVM embolization several embolic agents have been used including Histoacryl glue, polyvinyl alcohol particles (PVA), Gelfoam and coils. The objective of this study was to determine the efficacy of Uterine Artery Embolization in patients with bleeding acquired AVMs.

## Methods

This was a Prospective study where 225 patients referred to the department of Interventional Radiology for uterine artery embolization between July 2015 - April 2022 and 375 embolization procedures were done. The average age was 35.8 years. Serial β-human chorionic gonadotropin (hCG) levels were measured to exclude gestational trophoblastic neoplasia. Uterine artery embolizations were performed with use of standard 5F Robertson Uterine Curve catheter (RUC) and 2.7F Progreat microcatheter when necessary. Embolic agents used in the 375 procedures included Histoacryl glue only (n = 326), PVA particles and glue (n = 29), PVA particles (n = 5), Gelfoam (n = 5), coils (n = 4), PVA particles and coils (n = 3), glue and Gelfoam (n = 2), and glue and coils (n = 1) ( Table 1 ). Outcomes assessed were cessation of bleeding, persistence or resolution of the AVM, complications and pregnancy after embolization. These were assessed by chart, laboratory and imaging reviews. For all patients, clinical assessment was performed and informed consent was obtained before the procedure. Diagnosis was made based on clinical, imaging findings on Doppler and MRI examinations. Method of collection of data includes Brief clinical history and examination, fluoroscopic guided uterine artery embolization interventions, relevant biochemical investigations, and post procedural follow up. All the participants provided written informed consent.

**Table 1:** Embolic agents used

Embolic agents used	Number (n)	%
Histoacryl glue	326	86.9
PVA, Histoacryl glue	29	7.7
PVA	5	1.3
Gelfoam	5	1.3
Coils	4	1.0
PVA, Coils	3	0.8
Histoacryl glue, Gelfoam	2	0.5
Histoacryl glue, Coils	1	0.2
Total	375	

Abbreviation: PVA, Polyvinyl Alcohol.

## Embolization Technique

Procedure done under strict aseptic conditions, local anaesthesia (2% lignocaine). Anaesthetist was involved throughout the procedure. The Right Common Femoral Artery was accessed using 20G puncture needle and 5F Radifocus Introducer Sheath (Terumo) was placed by seldinger single puncture technique. Under fluoroscopic guidance using 5F 90cm RUC catheter (Merit Medical) and 0.032 150cm angled tip guide wire (Terumo) combination, the 0.032 guide wire was negotiated into contralateral external iliac artery (left). The RUC catheter was pushed into left external iliac artery. The guide wire is withdrawn into the RUC catheter. The RUC catheter was pushed up into abdominal aorta there by forming the curve. Left internal iliac artery was accessed using RUC catheter and 0.032 guide wire combination and angiogram demonstrated tortuous vessels supplying the AVM arising from the left uterine artery. The left uterine artery was selectively catheterized using 5F RUC catheter or by 2.7F 130cm Progreat Microcatheter (Terumo). The angiogram showed tortuous vessels supplying the AVM arising from the left uterine artery with drainage into a large venous channel. The AVM was embolized using Histoacryl glue (Braun) or PVA particles (Merit Medical 500-710 microns) and Histoacryl glue or PVA particles or Gelfoam (Spongostan Ethicon) or coils (Cook MicroNester Embolization coil) or PVA particles and coils or Histoacryl glue and Gelfoam or Histoacryl glue and coils. Post embolization angiogram showed Histoacryl glue cast within the branches of left uterine artery with no significant flow into the AVM. Similarly, the right uterine artery was accessed using a 5F RUC catheter or by 2.7F Progreat Microcatheter and the AVM was embolized. Check angiogram showed significant reduction of flow or stasis into the AVM. The introducer sheath was removed and compression bandage was applied. Post uterine AVM embolization all patients are advised bedrest with lower limb movement restriction and were treated with antibiotics (Ciprofloxacin, Amoxycillin + Clavulanic acid), analgesics, intravenous fluids and with supportive care. Patients were advised post procedural four weeks follow up with USG Abdomen, MRI Pelvis and relevant biochemical investigations.

## Results

**Fig. 1:**
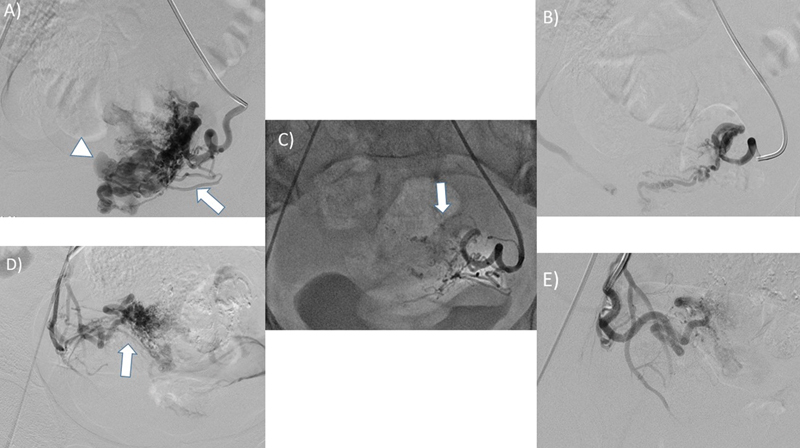
A 22 year young female patient with history of previous caesarian section presenting with recurrent per vaginal bleeding following D&C. (A and D) Bilateral uterine arteries were accessed using a 5F RUC catheter. The angiogram demonstrated tortuous vessels supplying the AVM (arrow) arising from the bilateral uterine arteries with drainage into a large venous channel (arrow head). (C) The AVM was embolized using PVA particles (500-710micron), followed by histoacryl glue which formed casts (arrow) within the network of arteries arising from the bilateral uterine arteries. (B and E) Repeat angiogram showed significant reduction of flow into the AVM. D&C: Dilatation and Curettage, AVM: Arteriovenous Malformation, RUC: Robertson Uterine Curve, PVA: Polyvinyl Alcohol.

**Fig. 2:**
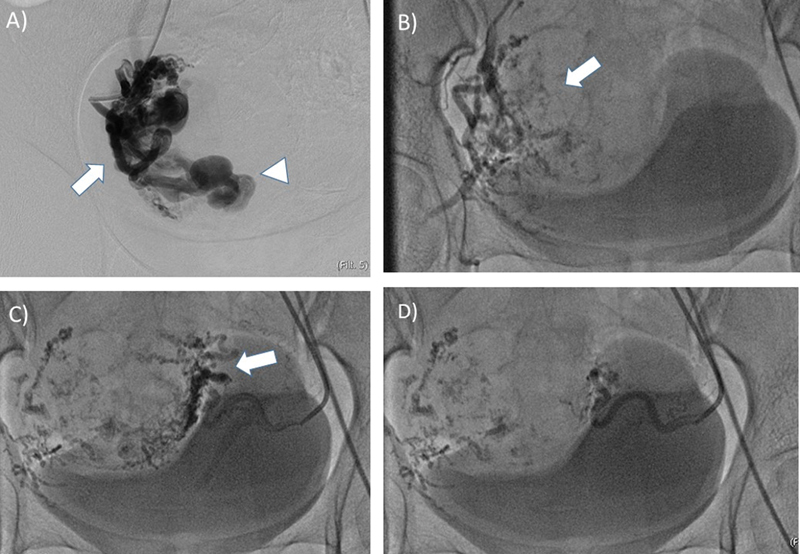
A 25 year young female patient presenting with menorrhagia and urinary incontinence, diagnosed with uterine AVM. (A) Using a 5F RUC catheter and 0.032 guide wire combination, right uterine artery was selectively catheterized and angiogram showed a large AVM (arrow) with drainage into a large venous channel (arrow head). (B) The AVM was embolized using histoacryl glue which formed casts (arrow) within the network of arteries. This was followed by embolization using 500-710micron PVA particles till it achieved stasis. (C) Similarly the left uterine artery was accessed using a 5F RUC catheter. The angiogram demonstrated tortuous vessels supplying the AVM (arrow). (D) The capillary network was embolized using 500-710micron PVA particles till the artery showed stasis in flow. AVM: Arteriovenous Malformation, RUC: Robertson Uterine Curve, PVA: Polyvinyl Alcohol.

**Fig. 3:**
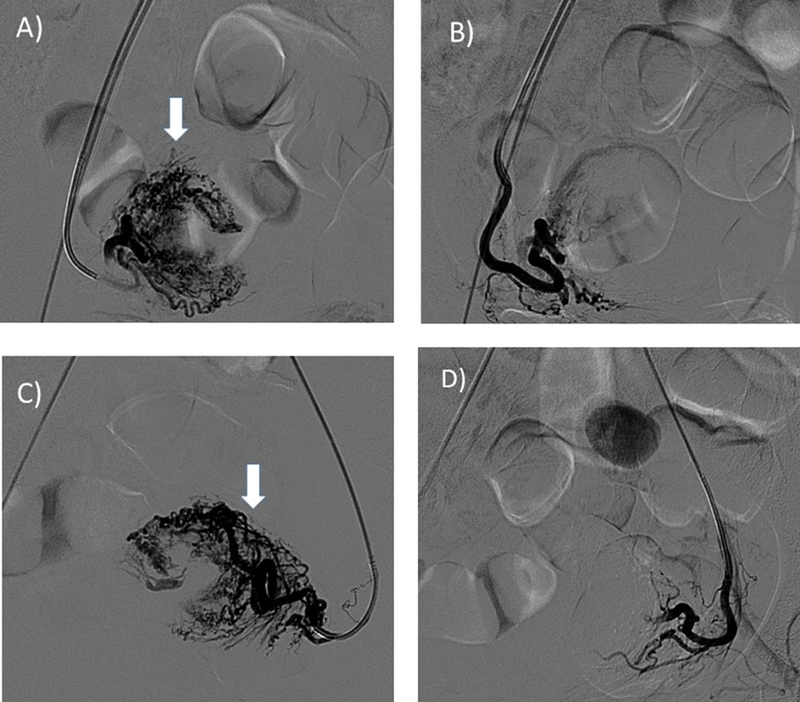
A 19-year-young woman with intermittent vaginal bleeding due to uterine AVM. (A and C) Selective catheterization of bilateral uterine arteries with 5F RUC catheter and angiogram shows tangle of blood vessels fed by Uterine Arteries (arrow). (B and D) Post Embolization angiogram with histoacryl glue shows significant reduction in Arterio-Venous shunting. AVM: Arteriovenous Malformation, RUC: Robertson Uterine Curve.

A total of 225 patients are included in our study (mean age, 35.8 years; age range, 22-43 years). Fourth decade individuals are most commonly effected. All 225 patients had previously undergone gynaecological procedures or obstetric events, such as dilatation and curettage (D&C) (n = 143) or delivery (n = 53) or abortion (n = 29) ( Table 2 ). Presenting symptoms were intermittent or progressive vaginal bleeding. The mean time interval between these obstetric events and symptom presentation was 7 weeks. A total of 375 embolization procedures were performed in 225 patients. Early venous drainage from the AVM to pelvic veins was demonstrated in all patients ( Fig. 1 ). 90 patients required repeat embolization for recurrence of bleeding ( Table 3 ). 3 of these patients underwent embolization six times over a median period of 44 months (range, 3-88 months), 6 of these patients underwent embolization thrice over a median period of 35 months (range, 4-74 months), 76 of these patients underwent embolization twice where the median time to second embolization was 21 months (range, 3–36 months). The embolic material used most often was Histoacryl glue in 358 procedures ( Fig. 2 ). 240 procedures were performed on an elective basis and 35 were performed on an emergent basis. Technical success was defined as the complete disappearance of angiographic staining of the AVM on post-embolization angiography and the absence of any procedure related complications ( Fig. 3 ). Clinical success was defined as immediate resolution of vaginal bleeding without symptom recurrence and resolution of the AVM on subsequent imaging studies. Technical success was achieved in 100% of cases. The clinical success rate was 92%. Vaginal bleeding was controlled in 222 of 225 patients and three patients underwent a hysterectomy. Recovery to normal menstrual cycle was seen in all 222 patients with clinical success within one or two months. Duplex ultrasound evaluation performed three days after embolization showed ultrasonographic success in all patients. 84 of the 225 patients conceived following embolization. 60 patients had uneventful intrauterine pregnancies carried to term. 15 woman had an induced abortions. Nine woman went into premature labor and in that four newborns died 1 week later. The longest delay between embolization and pregnancy was 3 years and the shortest was seven weeks. The mean time period between embolization and delivery was 21.3 months. The 210 patients who underwent successful embolization had no recurrence of bleeding at a median follow-up of 53 months (range, 5-122 months) after treatment. 15 patients were eventually lost to follow-up. One minor complication (0.4%) of non-flow-limiting dissection of the internal iliac artery occurred. There were no cases with uterine necrosis, non target embolization. No other side effects, either early or delayed, were documented as a result of the embolization procedure.

**Table 2:** 

Associated clinical history	Number	%
D&C	143	63
Delivery	53	24
Abortion	29	13
Total	225	

Abbreviations: D&C, dilation and curettage.

**Table 3:** 

No. of Patients	No. of Repeat Embolizations	Median Period (months)
76	2	21
6	3	35
3	6	44

## Discussion

Vascular malformations of the uterus are rare and potentially life threatening lesions. AVMs have been described in women of all ages, but predominantly among women of childbearing age. Uterine AVM can be congenital or acquired. Congenital uterine AVMs have multiple feeding arteries, a central tangle of vessels, and numerous large draining veins; these result from abnormal embryologic development of primitive vascular structures and tend to invade the surrounding structures. 11 12 13 14 Most congenital uterine AVMs are isolated anomalies, but can occur in association with AVMs at other sites. 15 16 Acquired uterine AVM is the predominant type of uterine AVM. It consists of multiple small arteriovenous fistulas between intramural arterial branches and the myometrial venous plexus, tends to have single or bilateral uterine artery feeders without an extrauterine arterial supply, and does not have a characteristic nidus. 17 18 19 20 Acquired Uterine AVM has been reported after abortion, caesarean section, direct uterine trauma like D&C and gestational trophoblastic disease etc.

During a normal pregnancy, utero-placental arteries are invaded by the trophoblast during the first two trimesters, which results artery enlargement and decreased resistance followed by re-endothelialisation during the third trimester. In subinvolution of placental bed vessels, uteroplacental arteries maintain characteristics similar to those of the first two trimesters. Some authors suggest that low flow uterine AVMs may be caused by subinvolution of placental bed vessels. 21 22 For normal pregnancies, there is an arteriovenous network in the myometrium that remains about 48 hours after delivery, 23 which may be a cause of uterine AVMs, specifically for high-flow AVMs. Patients with Uterine AVM can present with the following symptoms like menorrhagia, throbbing discomfort in the lower abdomen, urinary frequency or incontinence, dyspareunia, strong pelvic pulsations after exercise, systemic hypotension caused by blood pooling within the AVM, and even cardiac failure.

Clinical diagnosis of uterine AVM is often difficult and requires a high index of suspicion. Currently, transvaginal USG with doppler is the imaging modality of choice. Typical findings includes tortuous anechoic spaces in the myometrium which on colour doppler shows high-flow velocity with low resistance and mixing of arterial, venous waveforms where AVM drains into a large low-pressure venous pool **.**

Transcatheter arterial embolization is a minimally invasive treatment option with potential to preserve fertility because it does not seem to interfere with the menstrual cycle or pregnancy. 2 24 25 The typical finding of Uterine AVM by angiography is a high arterial flow with early venous filling. A single direct fistulous communication to the venous structures may be identified. Compared with surgical procedures, transcatheter arterial embolization is a safe and effective treatment option which includes low procedure related complication rates and shorter hospitalization. We have reported that treatment of uterine AVMs with arterial embolization has a high technical and clinical success rate. Our results demonstrate that this is effective and safe for the treatment of haemorrhagic complications of uterine AVM and may further preserve future fertility **.** Once the diagnosis of uterine AVM is made, embolization can be considered for anaemic or hemodynamically unstable patients. 26 Conversely, women who present with only one episode of metrorrhagia and who are hemodynamically stable should be monitored. 27 This is based on the hypothesis that uterine AVMs caused by subinvolution of the placental bed can have spontaneous involution. Curettage does not convey any risk for hemodynamically stable patients. On the opposite, hemodynamically unstable women should have an arteriogram and further embolization because curettage is at high risk in women with uterine AVMs. Embolization of the uterine arteries has not been associated with uterine infarction because of the presence of a rich collateral vascular network within the pelvis. 28

Although repeat embolization for recurrent bleeding may be required, hysterectomy can be avoided and fertility preserved, which is extremely important for this group of patient **s.** In our study 90 patients required repeat embolization for recurrence of bleeding. 3 of these patients underwent embolization six times, 6 of these patients underwent embolization thrice, 76 of these patients underwent embolization twice.

Our results demonstrate that Histoacryl glue is effective, safe and most commonly used embolic agent for the treatment of haemorrhagic complications due to uterine AVM and further preserves future fertility **.** It is an embolic agent that enables controlled and permanent obliteration of the AVM. It is a liquid ester that polymerizes rapidly in the presence of ionic substances like blood or saline. Histoacryl glue is mixed with lipiodol at a ratio ranging from 1:1 or 1:2. 0.1ml of this embolic agent mixture is injected followed by 25% w/v dextrose per injection. Other agents such as PVA particles, Gelfoam, coils are also used for embolization, especially when the target of embolization is more distal and further catheter advancement is difficult or impossible.

Failure of embolization therapy can be managed with hysterectomy 19 29 or with uterine artery and ovarian ligament ligation when uterine preservation is desired. 30 In our study three patients underwent hysterectomy as vaginal bleeding is not controlled after uterine artery embolization.

After successful embolization of a uterine AVM, hypovascularity of involved areas could, in theory, affect placentation and foetal growth; yet, several successful intrauterine pregnancies after transcatheter arterial embolization of uterine AVMs have been reported 20 31 32 including a successful twin pregnancy, 33 which suggests that adequate collateral blood supply can develop to support a full-term pregnancy. Normal placental blood flow has been documented after previous transcatheter arterial embolization to treat uterine AVM. 20 In the study by O'Brien et al., 34 normal menstrual cycles returned 2 months after transcatheter arterial embolization, and 5 patients became pregnant. In our study, we observed 84 patients conceived following embolization. 60 patients had uneventful full term pregnancy. Normal menstrual cycle was seen in 222 patients with clinical success within one or two months. Of note, our fertility rate is consistent with those reported in other studies. 8 35 Przybojewski and Sadler 36 reported a novel image guided management of a uterine AVM after failed transcatheter arterial embolization in which they directly injected embolization material into the nidus of the AVM under ultrasound guidance and fluoroscopy after exposing the uterus surgically; the patient had a successful term pregnancy afterward.

With experienced operators, transcatheter arterial embolization is generally safe. Minor complications including hematoma, urinary tract infection, retention of urine, and vessel or nerve injury at the vascular puncture site are common and require only mild supportive care or careful observation. 37 Varying degrees of pelvic pain are also common in the immediate post embolization period. Pain after transcatheter arterial embolization is probably due to ischemia produced by the embolization procedure, usually peaks on the first day after the procedure, responds well to analgesic therapy, and resolves in about a week. 38 Uterine necrosis is another rare life-threatening complication that mandates prompt treatment with antibiotics and hysterectomy. Loss of ovarian function can rarely develop after uterine artery embolization, in particular in women older than 45 years because of their more abundant uterine ovarian arterial anastomoses compared with younger women. 39 40 Other serious potential complications of transcatheter arterial embolization may include perianal skin sloughing, uterovaginal and rectovaginal fistulas, neurologic deficits in the lower extremities, deep venous thrombosis, and pulmonary embolism. 33 41 42 43 In our study, one minor complication of non-flow-limiting dissection of the internal iliac artery occurred. It was subsequently embolized using microcoils. Otherwise there were no cases with uterine necrosis, non target embolization and other serious complications in our patients. Our study has limitations like 15 patients were eventually lost to follow-up.

## Conclusion

Uterine AVM is a rare but potentially serious cause of abnormal vaginal bleeding. Diagnosis should be considered in all patients of reproductive age who have abnormal vaginal bleeding and negative β-hCG test results. Transvaginal ultrasonography is the imaging method of choice. Colour Doppler imaging should be used routinely to enable the correct diagnosis. Transcatheter uterine artery embolization is an excellent treatment option with a low complication rate, high success rate and further preserves future fertility. It is a safe and effective treatment for severe bleeding uterine vascular malformations.
